# ZDHHC11 Suppresses Zika Virus Infections by Palmitoylating the Envelope Protein

**DOI:** 10.3390/v15010144

**Published:** 2023-01-02

**Authors:** Dingwen Hu, Haimei Zou, Weijie Chen, Yuting Li, Ziqing Luo, Xianyang Wang, Dekuan Guo, Yu Meng, Feng Liao, Wenbiao Wang, Ying Zhu, Jianguo Wu, Geng Li

**Affiliations:** 1 State Key Laboratory of Virology, College of Life Sciences, Wuhan University, Wuhan 430072, China; 2 The Second Affiliated Hospital, Guangzhou University of Chinese Medicine, Guangzhou 510120, China; 3 Guangdong Provincial Key Laboratory of Virology, Institute of Medical Microbiology, Jinan University, Guangzhou 510632, China; 4 Laboratory Animal Center, Guangzhou University of Chinese Medicine, Guangzhou 510006, China; 5 Medical Research Center, Guangdong Provincial People’s Hospital, Guangdong Academy of Medical Sciences, Guangzhou 510080, China

**Keywords:** Zika virus, palmitoylation, 2-BP, envelope protein, ZDHHC11

## Abstract

Zika virus (ZIKV) is an RNA-enveloped virus that belongs to the *Flavivirus genus*, and ZIKV infections potentially induce severe neurodegenerative diseases and impair male fertility. Palmitoylation is an important post-translational modification of proteins that is mediated by a series of DHHC-palmitoyl transferases, which are implicated in various biological processes and viral infections. However, it remains to be investigated whether palmitoylation regulates ZIKV infections. In this study, we initially observed that the inhibition of palmitoylation by 2-bromopalmitate (2-BP) enhanced ZIKV infections, and determined that the envelope protein of ZIKV is palmitoylated at Cys308. ZDHHC11 was identified as the predominant enzyme that interacts with the ZIKV envelope protein and catalyzes its palmitoylation. Notably, ZDHHC11 suppressed ZIKV infections in an enzymatic activity-dependent manner and ZDHHC11 knockdown promoted ZIKV infection. In conclusion, we proposed that the envelope protein of ZIKV undergoes a novel post-translational modification and identified a distinct mechanism in which ZDHHC11 suppresses ZIKV infections via palmitoylation of the ZIKV envelope protein.

## 1. Introduction

The Zika virus (ZIKV) was initially detected in the Rhesus macaque in Uganda in 1947, and was endemic in the Americas from 2014 to 2015 [1]. ZIKV belongs to the *Flavivirus genus*, and, like the dengue virus, the clinical symptoms of ZIKV infections are self-limited and primarily include fever, headache, and muscle aches, among other symptoms [2]. However, ZIKV infections are also linked to microcephaly in neonates [3], Guillain–Barré syndrome in adults [4], and even male infertility [5], which has raised global concerns. Similar to other flaviviruses, ZIKV is an enveloped virus with a single-stranded RNA genome of approximately 11 kb [6]. Following infection, the viral genome is injected into susceptible host cells and is translated into a large polyprotein, which is cleaved into three structural proteins, namely, capsid, envelope, and pr-Membrane proteins, and seven non-structural (NS) proteins, namely, NS1, NS2A, NS2B, NS3, NS4A, NS4B, and NS5 [6]. The NS proteins are primarily associated with genome replication, immune evasion, and pathological damage [7,8,9], while the structural proteins are mainly involved in receptor binding, genome packaging, and virion assembly [10].

The envelope protein of ZIKV is a crucial multifunctional protein that forms the viral surface along with the Membrane protein, and facilitates viral entry by binding to cell surface receptors and promotes membrane fusion [11]. The envelope protein is the primary viral protein that induces host neutralizing antibodies [11]. The functions of the envelope protein are therefore strictly regulated by various post-translational modification systems of host cells. The TRIM7-mediated ubiquitination of the envelope protein facilitates viral entry and pathogenesis, and thus promotes viral infections; however, the host deubiquitination system catalyzes deubiquitination of the envelope protein via a series of ubiquitin-specific proteases, and thus exerts an antiviral effect [12]. Furthermore, a previous study reported that glycosylation of the envelope protein of ZIKV at Asn154 mediates receptor binding [13].

Palmitoylation is a reversible protein post-translational modification in which a palmitate moiety is attached to the cysteine residues of target proteins, and contributes to protein trafficking, stability, and functionality [14]. The palmitoylation of proteins is mediated by a series of enzymes that contain a zinc finger Asp–His–His–Cys (ZDHHC) motif, which are named ZDHHC1 to ZDHHC9 and ZDHHC11 to ZDHHC24 [14]. It has been demonstrated that the ZDHHC11 protein anchors to the endoplasmic reticulum (ER) and regulates the oligomerization of TRFA6 to induce the activation of NF-kB [15]; ZDHHC11 also promotes MITA-IRF3 interactions for facilitating the immune response following infections with DNA viruses [16].

It has been identified that the palmitoylation of proteins plays an essential role in the regulation of tumorigenesis, immune response, and inflammatory reactions [17,18,19]. Viral proteins also undergo palmitoylation for regulating viral infections. For instance, palmitoylation of the spike protein of severe acute respiratory syndrome coronavirus 2 (SARS-CoV-2) is essential for viral infectivity [20,21], while palmitoylation of the hemagglutinin (HA) protein of influenza virus is crucial for membrane fusion [22,23]. However, it remains to be investigated whether the proteins of ZIKV are modified by palmitoylation.

The findings of this study demonstrated that the presence of a palmitoylation inhibitor enhanced ZIKV infection and that the envelope protein of ZIKV is palmitoylated at Cys308. ZDHHC11 was identified as the predominant enzyme that interacts with the envelope protein and mediates its palmitoylation. Notably, the study revealed that the overexpression of ZDHHC11 suppressed ZIKV infection, while ZDHHC11 knockdown enhanced viral infection.

## 2. Materials and Methods

### 2.1. Cell Lines and Cultures

HEK293T cells, Vero cells, and C6/36 cells were purchased from the American Type Culture Collection (ATCC), and U251 cells were purchased from China Center for Type Culture Collection (CCTCC). BHK-21 cells were kindly gifted by Dr. Cheng-Feng Qin of the Beijing Institute of Microbiology and Epidemiology, China. The HEK293T cells, Vero cells, U251 cells, and BHK-21 cells were maintained in Dulbecco’s modified Eagle’s medium (DMEM) supplemented with 10% fetal bovine serum (FBS) and 1% Penicillin-Streptomycin at 37 °C in a humidified incubator containing 5% CO_2_. The C6/36 cells were maintained in RPMI 1640 medium supplemented with 10% FBS and 1% Penicillin-Streptomycin at 30 °C in a humidified incubator containing 5% CO_2_.

### 2.2. Antibodies and Reagents

Antibodies against Flag (F3165, 1:2000) and HA (H6908, 1:5000) were purchased from Sigma (Burlington, MA, USA). Antibodies against the envelope protein of ZIKV (GTX133314, 1:1000), NS1 protein of ZIKV (GTX133307, 1:1000), glyceraldehyde-3-phosphate dehydrogenase (GAPDH; GTX100118, 1:5000), and ZDHHC11 (GTX106800, 1:1000) were purchased from GeneTex (Hsinchu City, Taiwan, P.R.C). For the immunofluorescence studies, antibodies against the envelope protein of flaviviruses (ab214333, 1:100) were purchased from Abcam (Shanghai, China). N-ethylmaleimide (E3876), hydroxylamine hydrochloride (255580), and 2-bromopalmitate (2-BP; 21604) were obtained from Sigma (Burlington, MA, USA). BMCC-biotin (21900) was purchased from Thermo Fisher Scientific (Waltham, MA, USA). Streptavidin-horseradish peroxidase (M00091) was purchased from Genscript (Piscataway, NJ, USA). Fluorescein (FITC)-conjugated AffiniPure goat anti-mouse immunoglobulin (Ig)G (H+L) (SA00003-1) and Cy3-conjugated AffiniPure goat anti-rabbit IgG (H+L) (SA00009-2) were purchased from ProteinTech (Rosemont, IL, USA).

### 2.3. Viruses

Information pertaining to ZIKV and protocols used for the culture of ZIKV in this study have been described in our previous studies [24].

### 2.4. Plasmid Construction

The Flag-tagged ZDHHC1–ZDHHC24 expression plasmids were purchased from WZ Biosciences (Columbia, MD, USA). The expression plasmids for the ZIKV capsid, prMembrane/membrane, and envelope protein were constructed as described in our previous study [24].

### 2.5. RNA Interference Studies

Short interfering RNAs (siRNAs) targeting the human ZDHHC11 mRNA were designed and synthesized by HippoBio and transfected to U251 cells using lipofectamine 2000 (Invitrogen, CA, USA, 11668019), according to the manufacturer’s instructions. The following sequences were used for the RNA interference studies:

si-ZDHHC11-1: 5′-CGCAAAGAAGAGAGUUCAA-3′

si-ZDHHC11-2: 5′-GGUAUGAAGAUGUCAAGAA-3′

si-ZDHHC11-3: 5′-GCCAAGAAGAUGACCACCUUU-3′

### 2.6. Immunoblotting and Immunoprecipitation Studies

The cells were lysed in lysis buffer (150 mM NaCl, 50 mM Tris–HCl, 5 mM EDTA, 10% glycerol, and 1% Triton X-100) with a complete protease inhibitor cocktail. Western blot analysis was performed using the indicated antibodies. For immunoprecipitation, each lysate was incubated overnight with the indicated antibody with rotation at 4 °C, following which protein A/G agarose (Pierce) was added for capturing the antibody complex. The beads were finally washed thrice with lysis buffer and eluted through incubation at 100 °C for 10 min in 70 μL of 2× loading buffer. The eluted samples were assayed with immunoblotting.

### 2.7. Quantitative Real-Time Polymerase Chain Reaction (PCR)

The total RNA was extracted with Trizol reagent and quantified using real-time PCR as previously described [24]. The following primers were used for PCR:

ZIKV-forward: 5′-GGTCAGCGTCCTCTCTAATAAACG-3′

ZIKV-reverse: 3′-GCACCCTAGTGTCCACTTTTTCC-5′

GAPDH-forward: 5′-ATGACATCAAGAAG GTGGTG-3′

GAPDH-reverse: 3′-CATACCAGGAAATGAGCTTG-5′

ZDHHC11-forward: 5′-GATCTTCTCGTTCCACCTCGT-3′

ZDHHC11-reverse: 3′-TGTGCATGTTTTGATCTGTCGAA-5′

### 2.8. Immunofluorescence Analysis

The cells were washed with phosphate-buffered saline (PBS), supplemented with 0.1% bovine serum albumin (BSA), and subsequently fixed with 4% paraformaldehyde for 15 min. The cells were washed thrice with PBS, supplemented with 0.1% BSA, permeabilized with 0.1% Triton X-100 for 5 min at room temperature, and incubated with blocking solution (PBS, 5% BSA) for an additional 30 min. The cells were subsequently incubated overnight with the primary antibody at 4 °C, washed thrice with PBS, and further incubated with the secondary FITC-conjugated antibody or Cy3 for 30 min. The slides were washed thrice with PBS and mounted onto glass coverslips using Prolong Gold Antifade Reagent with DAPI. The processed slides were imaged using a Zeiss LSM710 confocal microscope.

### 2.9. Acyl-Biotin Exchange (ABE) Assay

The ABE assay was performed for determining palmitoylation, as previously described [17]. Briefly, the cells were lysed using lysis buffer, containing 50 μM N-ethylmaleimide, and the target proteins were immunoprecipitated by adding the corresponding antibody. The immunoprecipitated complexes were resuspended with 1 M hydroxylamine hydrochloride buffer for 1 h at room temperature, and subsequently washed five times with lysis buffer (pH = 6.2). The beads were then resuspended with BMCC-biotin (5 μM) buffer for 2 h at room temperature and subsequently washed five times. The complexes were analyzed with sodium dodecyl-sulfate polyacrylamide gel electrophoresis (SDS-PAGE) and detected using the indicated antibody.

### 2.10. Statistics Analysis

All the data were analyzed using GraphPad Prism v8.4.0. Statistical significance was determined using Student’s *t* test (*, *p* < 0.05; **, *p* < 0.01; ***, *p* < 0.001).

## 3. Results

### 3.1. 2-BP Enhances ZIKV Infection in Vero Cells

**Figure 1:**
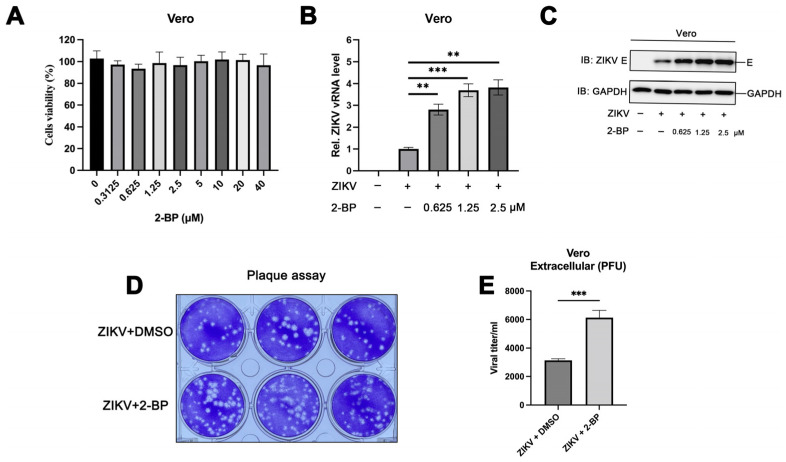
The palmitoylation inhibitor, 2-BP, enhances ZIKV infection in Vero cells. (**A**) Vero cells were treated with DMSO or indicated concentrations of 2-BP for 24 h, following which the cell viability was detected using CCK-8 assays. (**B**–**E**) Vero cells were treated with DMSO or indicated concentrations of 2-BP for 6 h and subsequently infected with ZIKV (multiplicity of infection (MOI) = 1) using incubation for 24 h. (**B**) The levels of viral RNA were measured with qPCR. (**C**) The content of viral proteins was detected using immunoblotting studies, and (**D**,**E**) the ZIKV titer in the supernatants was calculated with plaque assays. (**, *p* < 0.01; ***, *p* < 0.001).

We have previously reported several host factors that target Zika virus envelope protein ubiquitination [12], a kind of dynamic protein post-translational modification, to inhibit viral infection [24,25]. Palmitoylation is an important post-translational modification of proteins, and viral proteins undergo palmitoylation during viral infections. In this study, we initially investigated the role of palmitoylation in ZIKV infections. The palmitate analogue, 2-BP, is widely used in studies on protein palmitoylation; it irreversibly inhibits the enzymatic activity of all palmitoyltransferases and serves as a non-selective inhibitor of protein palmitoylation [26]. To this end, we first evaluated the cytotoxicity of 2-BP at various concentrations in Vero cells using CCK-8 assays, and the results demonstrated that 2-BP was not cytotoxic at concentrations below 40 μM (Figure 1A). The ZIKV-infected Vero cells were subsequently treated with 2-BP. Interestingly, the results of real-time quantitative PCR and immunoblotting studies demonstrated that the levels of viral RNA and expression of viral proteins increased significantly following treatment with 2-BP (Figure 1B,C). The findings were consistent with the results of plaque assays, which revealed that treatment with 2-BP enhanced ZIKV infection in Vero cells (Figure 1D,E). These findings suggested that the inhibition of palmitoylation promotes ZIKV infections, and that the palmitoylation of viral proteins suppresses ZIKV infection.

### 3.2. The ZIKV Envelope Protein Is Palmitoylated at Cys308

**Figure 2:**
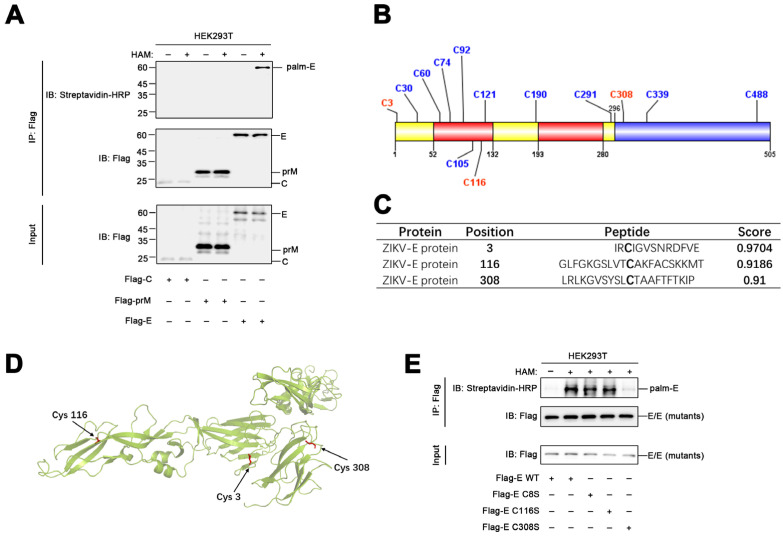
The envelope protein of ZIKV is palmitoylated at Cys308. (**A**) HEK293T cells were transfected with a plasmid encoding the ZIKV capsid (C), prMembrane (prM), and envelope (E) proteins for 24 h, lysed for ABE assays, and detected using immunoblotting with the indicated antibodies. (**B**) Schematic depicting all the 13 cysteine residues in the structure of the envelope protein. (**C**) Possible palmitoylation sites in the envelope protein of ZIKV were predicted using the GPS-Palm system. (**D**) The predicted cysteine residues are highlighted in the structure of the envelope protein. (**E**) HEK293T cells were transfected with plasmids encoding the WT or mutated envelope proteins for 24 h, lysed for ABE assays, and the palmitoylation states were detected using immunoblotting with the indicated antibodies.

In order to determine the mechanism by which protein palmitoylation regulates ZIKV infections, we attempted to determine whether ZIKV proteins undergo palmitoylation in host cells. ABE assays were performed using HEK293T cells expressing the structural proteins of ZIKV, including the capsid, pre-Membrane, and envelope proteins. The results demonstrated that the envelope protein underwent palmitoylation, while the capsid and pre-Membrane proteins were not palmitoylated (Figure 2A). Palmitoylation typically occurs at the cysteine residues of target proteins, and the envelope protein of ZIKV contains 13 cysteine residues (Figure 2B). In order to determine the specific residue of the ZIKV envelope protein that undergoes palmitoylation, we initially used the GPS-Palm palmitoylation site prediction system [27], which revealed that Cys3, Cys116, and Cys308 were the most likely sites for palmitoylation (Figure 2C). Of these, Cys3 is located in the central-barrel like Domain I (yellow), Cys116 is located in the elongated finger-like structure Domain II (red), and Cys308 is located in the immunoglobulin-like module Domain III (blue) (Figure 2B,D). These cysteine residues were subsequently substituted with a serine, and ABE assays were conducted in HEK293T cells overexpressing the wild-type (WT) or mutated envelope proteins. The results demonstrated that the palmitoylation level of only the Cys308Ser mutant was significantly reduced compared to that of the WT envelope protein (Figure 2E). The finding suggested that the envelope protein of ZIKV is palmitoylated at Cys308.

### 3.3. ZDHHC11 Mediates the Palmitoylation of the ZIKV Envelope Protein

**Figure 3:**
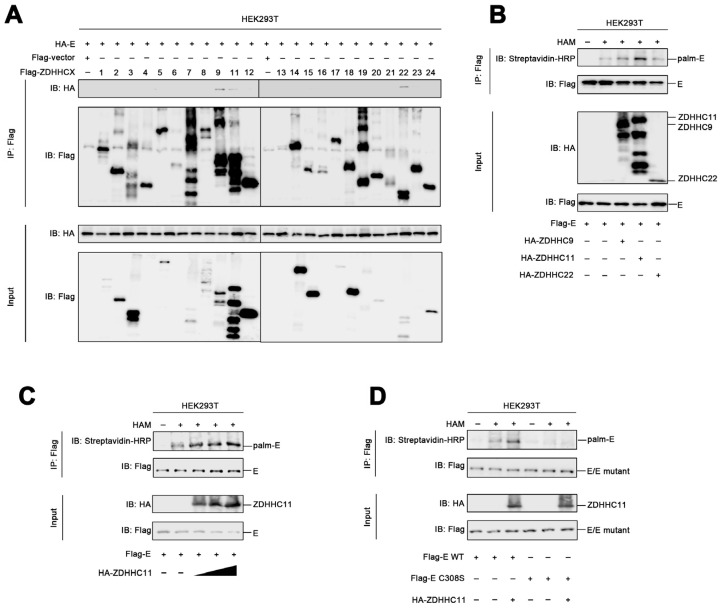
ZDHHC11 mediates the palmitoylation of the ZIKV envelope protein. (**A**) HEK293T cells were transfected with plasmids encoding the envelope protein and ZDHHCXs for 24 h, lysed for co-immunoprecipitation assays using the Flag antibody, and detected using immunoblotting studies with the indicated antibodies. (**B**) HEK293T cells were transfected with plasmids encoding the envelope protein, ZDHHC9, ZDHHC11, and ZDHHC22 for 24 h, lysed for ABE assays, and detected using immunoblotting with the indicated antibodies. (**C**) HEK293T cells were transfected with plasmids encoding the envelope protein and ZDHHC11 for 24 h, lysed for ABE assays, and detected using immunoblotting with the indicated antibodies. (**D**) HEK293T cells were transfected with plasmids encoding the WT or mutant envelope proteins and ZDHHC11 for 24 h, lysed for ABE assays, and detected using immunoblotting with the indicated antibodies.

Protein palmitoylation is generally catalyzed by a group of enzymes containing the conserved zinc finger Asp–His–His–Cys (ZDHHC) motif [14]. In order to determine the specific enzyme that catalyzes the palmitoylation of the envelope protein, we constructed expression plasmids encoding 23 palmitoyltransferases, including ZDHHC1 to ZDHHC9 and ZDHHC11 to ZDHHC24. HEK293T cells were subsequently transfected with the plasmids expressing the envelope protein and the ZDHHCs. The results of co-immunoprecipitation experiments revealed that ZDHHC9, ZDHHC11, and ZDHHC22 were associated with the envelope protein of ZIKV (Figure 3A), which suggested that ZDHHC9, ZDHHC11, and ZDHHC22 could be involved in the palmitoylation of the ZIKV envelope protein. In order to elucidate the specific enzyme that catalyzes the palmitoylation of the ZIKV envelope protein, HEK293T cells were transfected with plasmids expressing the envelope protein, ZDHHC9, ZDHHC11, and ZDHHC22, and subjected to ABE assays. The findings revealed that the level of palmitoylation of the ZIKV envelope protein was markedly enhanced by ZDHHC11 but not by ZDHHC9 and ZDHHC22 (Figure 3B). Additional ABE assays confirmed that the palmitoylation of the envelope protein was remarkedly induced when the expression of ZDHHC11 was increased (Figure 3C), indicating that ZDHHC11 mediated the palmitoylation of the ZIKV envelope. As aforementioned, the level of palmitoylation of the envelope protein reduced significantly when Cys308 was substituted to serine (Figure 2E), and ZDHHC11 failed to induce palmitoylation of the mutant Cys308Ser envelope protein (Figure 3D). Taken together, these findings demonstrated that ZDHHC11 mediated the palmitoylation of the ZIKV envelope protein at Cys308.

### 3.4. ZDHHC11 Interacts with the ZIKV Envelope Protein

**Figure 4:**
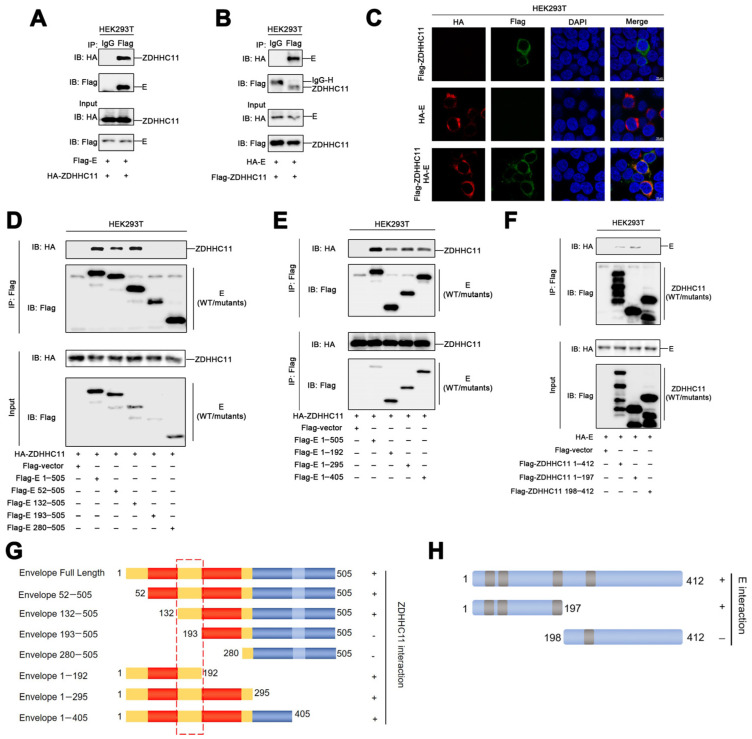
ZDHH11 interacts with the envelope protein of ZIKV. (**A**,**B**) HEK293T cells were transfected with plasmids encoding the envelope protein and ZDHHC11 for 24 h, lysed for co-immunoprecipitation assays with the indicated antibodies, and detected using immunoblotting with the indicated antibodies. (**C**) HEK293T cells were transfected with HA-E or Flag-ZDHHC11, or co-transfected with HA-E and Flag-ZDHHC11. The sub-cellular localizations of HA-E (red), Flag-ZDHHC11 (green), and the nuclear marker, DAPI (blue) were analyzed with CLSM. (**D**,**E**) HEK293T cells were transfected with plasmids encoding ZDHHC11 and the WT or truncated envelope proteins for 24 h, lysed for co-immunoprecipitation assays with the indicated antibodies, and detected using immunoblotting with the indicated antibodies. (**F**) HEK293T cells were transfected with plasmids encoding the envelope protein and ZDHHC11 or truncated ZDHHC11 for 24 h, lysed for co-immunoprecipitation assays with the indicated antibodies, and detected using immunoblotting with the indicated antibodies. (**G**) Diagrammatic representation of the full-length and truncated envelope protein. Domain I, II, and III are marked by yellow, red and blue. (**H**) Diagrammatic representation of the full-length and truncated ZDHHC11 enzyme.

In order to determine the palmitoylation of the envelope protein by ZDHHC11, co-immunoprecipitation experiments were performed using HEK293T cells transfected with plasmids expressing the ZIKV envelope protein and ZDHHC11. The results demonstrated that ZDHHC11 interacted with the envelope protein (Figure 4A), and that the ZIKV envelope protein co-immunoprecipitated with ZDHHC11 (Figure 4B). Confocal laser scanning microscopy (CLSM) indicated that the envelope protein and ZDHHC11 co-localized and formed large spots in the cytoplasm of HEK293T cells (Figure 4C). Based on structural analyses of the ZIKV envelope protein, we constructed seven plasmids encoding different truncated envelope proteins (Figure 4G) for identifying the specific region of the ZIKV envelope that binds to ZDHHC11. The results of co-immunoprecipitation assays revealed that, similar to the full-length ZIKV envelope protein (1–505 aa), the truncated 52–505 and 132–505 aa envelope proteins could interact with ZDHHC11; however, the truncated 193–505 and 280–505 aa envelope proteins did not interact with ZDHHC11 (Figure 4D). Additionally, three truncated envelope proteins (1–193, 1–296, and 1–406 aa) interacted with ZDHHC11 (Figure 4E). These results demonstrated that the partial Domain I region (132–193 aa) of the ZIKV envelope protein mediates the envelope-ZDHHC11 interactions (Figure 4G). The results of co-immunoprecipitation studies also demonstrated that the N-terminal region (1–197 aa) of ZDHHC11 is required for binding to the ZIKV envelope (Figure 4F,H). Taken together, these results suggested that ZDHHC11 binds to the Domain I region (132–193 aa) of the ZIKV envelope via its N-terminal region (1–197 aa) to subsequently catalyze the palmitoylation of the envelope protein.

### 3.5. Overexpression of ZDHHC11 Inhibits ZIKV Infection

**Figure 5:**
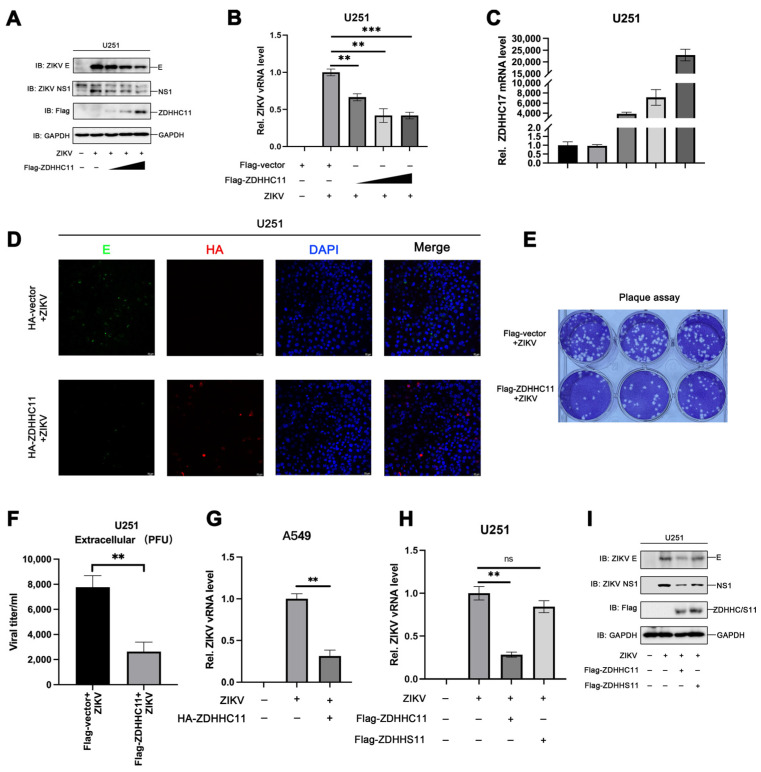
The overexpression of ZDHHC11 inhibits ZIKV infection. (**A**–**F**) U251 cells were transfected with plasmids encoding Flag-ZDHHC11 or control vector for 12 h and subsequently infected with ZIKV (MOI = 1) using incubation for 48 h. The content of viral proteins was detected by (**A**) immunoblotting and (**D**) confocal microscopy. The levels of (**B**) viral RNA and (**C**) ZDHHC11 mRNA were measured by quantitative PCR. (**E**,**F**) The titer of ZIKV in the supernatants was calculated using plaque assays. (**G**) A549 cells were transfected with plasmids encoding HA-ZDHHC11 or control vector for 12 h and subsequently infected with ZIKV (MOI = 1) using incubation for 48 h. The content of viral RNA was detected by qPCR. (**H**,**I**) U251 cells were transfected with plasmids encoding Flag-ZDHHC11, Flag-ZDHHS11, or the control vector for 12 h and subsequently infected with ZIKV (MOI = 1) using incubation for 48 h. (**H**) The levels of viral RNA were measured by quantitative PCR and (**I**) the content of viral proteins was detected using immunoblotting. (ns, no significance; **, *p* < 0.01; ***, *p* < 0.001).

The aforementioned results demonstrated that the ZIKV envelope protein is palmitoylated by ZDHHC11, and treatment with the palmitoylation inhibitor, 2-BP, promotes ZIKV infection. In this study, we also investigated the role of ZDHHC11 in ZIKV infections. The results demonstrated that the expression of the envelope protein and NS1 in ZIKV-infected U251 cells transfected with expression plasmids encoding ZDHHC11 was attenuated as the expression of ZDHHC11 increased (Figure 5A), while the levels of viral RNA reduced markedly as the ZDHHC11 mRNA levels increased (Figure 5B,C). These findings were consistent with the results of immunofluorescence staining, which revealed that the content of viral proteins decreased significantly in the presence of ZDHHC11 (Figure 5D). The viral titer in the supernatant of ZIKV-infected U251 cells was also determined and quantified using plaque assays, and the results demonstrated that ZIKV infection was dramatically attenuated following the overexpression of ZDHHC11 (Figure 5E,F). Consistent with this, the overexpression of ZDHHC11 also inhibited ZIKV infection in A549 cells (Figure 5G). We also generated an inactivated form of ZDHHC11 (ZDHHS11) by inducing a mutation in the DHHC motif, and evaluated its effect on ZIKV infection. The results of real-time quantitative PCR and immunoblotting studies demonstrated that the levels of viral RNA and the expression of viral proteins decreased significantly in the presence of ZDHHC11 but not ZDHHS11 in ZIKV-infected U251 cells (Figure 5H,I). The findings indicated that the enzymatic activity of ZDHHC11 is essential for the anti-ZIKV effect. Overall, these results suggested that the overexpression of ZDHHC11 inhibits ZIKV infection in an enzymatic activity-dependent manner.

### 3.6. ZDHHC11 Knockdown Enhances ZIKV Infection

**Figure 6:**
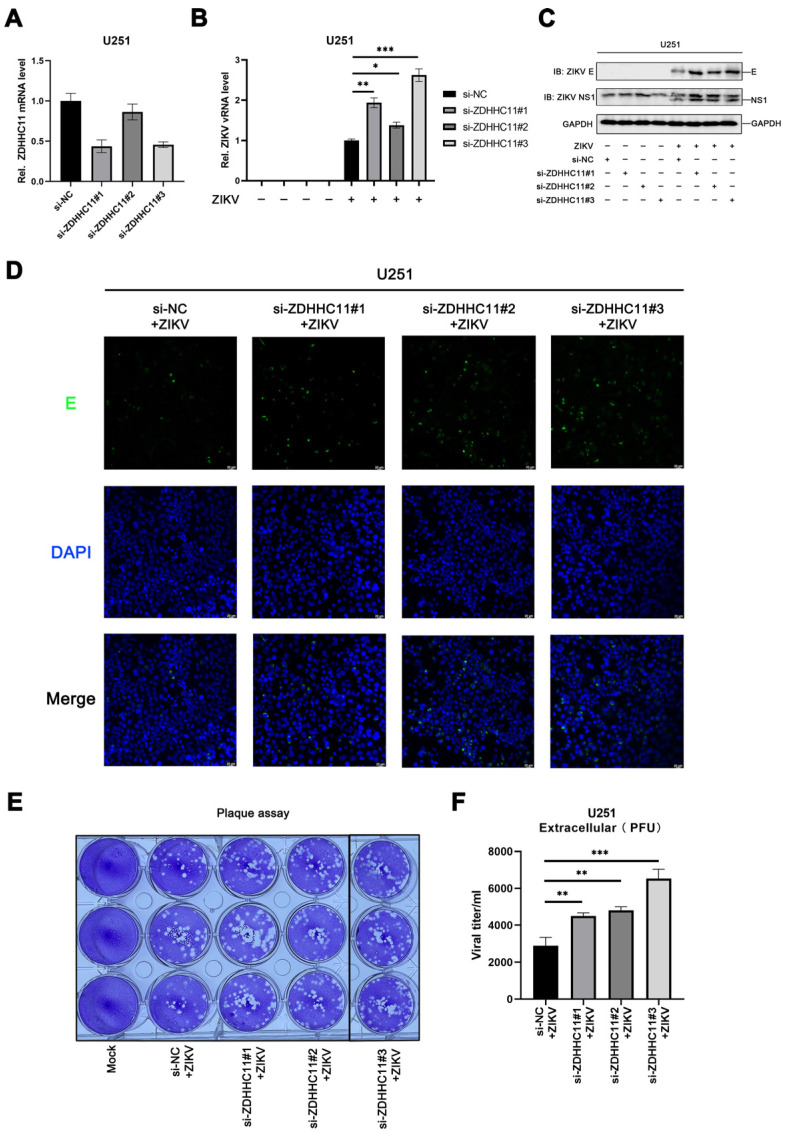
ZDHHC11 knockdown enhances ZIKV infection. U251 cells were transfected with anti-ZDHHC11 siRNA or the negative control, and subsequently infected with ZIKV (MOI = 1) using incubation for 24 h. The mRNA levels of (**A**) ZDHHC11 and (**B**) viral RNA were measured with quantitative PCR. The abundance of viral proteins was detected with (**C**) immunoblotting and (**D**) CLSM. (**E**,**F**) The ZIKV titer in the supernatants was calculated using plaque assays. (*, *p* < 0.05; **, *p* < 0.01; ***, *p* < 0.001).

To further confirm the role of ZDHHC11 in ZIKV infections, the endogenous ZDHHC11 in U251 cells was knocked down using an RNA interference strategy (Figure 6A). The levels of viral RNA and expression of viral proteins, including the envelope protein and NSP1, increased significantly in ZIKV-infected U251 cells, following transfection with anti-ZDHHC11 siRNA (Figure 6B,C). The results of CLSM also demonstrated that the ZIKV envelope protein content increased substantially following ZDHHC11 knockdown (Figure 6D). The viral titer in the supernatant of ZIKV-infected U251 cells subjected to siRNA transfection was detected and quantified using plaque assays. The results demonstrated that ZIKV infection increased significantly following ZDHHC11 knockdown (Figure 6E,F). These findings revealed that ZDHHC11 knockdown enhances ZIKV infection, and that ZDHHC11 has an anti-ZIKV effect.

## 4. Discussion

A comprehensive understanding of virus–host interactions is important for identifying the pathogenic mechanisms of viruses and developing novel therapeutic strategies. In this study, we observed that treatment with the palmitoylation inhibitor, 2-BP, enhanced ZIKV infection in Vero cells, which suggested that protein palmitoylation suppresses ZIKV infections. However, there are no reports on the palmitoylation of ZIKV proteins to date. In this study, we identified that the envelope protein of ZIKV undergoes palmitoylation, and subsequent computational and mutation analyses confirmed that the envelope protein is palmitoylated at Cys308. ZDHHC11 was identified as the predominant enzyme that catalyzes the palmitoylation of the envelope protein. We also found that ZDHHC11 binds to the Domain II region (132–193 aa) of the envelope protein via its N-terminal region (1–197 aa). The role of ZDHHC11 in ZIKV infection was investigated, and the results demonstrated that the overexpression of ZDHHC11 suppressed ZIKV infection in an enzymatic activity-dependent manner, while ZDHHC11 knockdown enhanced viral infections.

The envelope protein of flaviviruses plays crucial roles in viral structure, invasion, and assembly, and also serves as the primary antigen in inducing the production of host antibodies [11]. The functions of the envelope protein are tightly regulated by several post-translational modifications. The envelope proteins of several flaviviruses undergo N-linked glycosylation, in which a glycan is attached to the amide nitrogen of Asp [28]. It has been reported that the glycosylation of the envelope protein of ZIKV at Asn154 facilitates the expression and secretion of E ectodomain, production of virus like particle (VLP), and infectivity [29]. A previous study demonstrated that deficient glycosylation enhances viral entry, virion assembly, and the production of progeny viruses in C6/36 cells [30], but reduces pathogenesis in ifnar1^−/−^ mice [13,31]. A recent study reported that the ZIKV envelope protein undergoes K63-linked polyubiquitination at Lys38 and Lys281, which is catalyzed by the E3-ubiquitin ligase, TRIM7 [12]. Ubiquitination of the envelope protein promotes viral entry and pathogenesis, while deubiquitylation of the envelope protein by ubiquitin-specific proteases exerts antiviral effects [24,25]. The results of this study demonstrated that the ZIKV envelope protein undergoes palmitoylation, which is a novel post-translational modification of proteins. The palmitoylation of the envelope protein possibly serves as an antiviral strategy in host cells, as ZIKV infections were enhanced following treatment with the palmitoylation inhibitor, 2-BP. Subsequently, computational and mutation analyses confirmed that palmitoylation of the envelope protein occurs at Cys308. As flaviviral envelope proteins are highly homologous and have high amino acid sequence identity, it is likely that the envelope proteins of other flaviviruses are modified by palmitoylation. Understanding the relationship between palmitoylation and viral invasion, release, and occurrence of other post-translational modifications of viral proteins may provide better insights into the effects of palmitoylation on ZIKV.

Palmitoylation is a reversible post-translational modification of proteins that regulates the localization, stability, and functions of proteins by attaching palmitic acid to the cysteine residues of target proteins. Several studies have indicated that palmitoylation regulates the functions of coronavirus proteins. Previous studies have demonstrated that palmitoylation of the spike (S) protein of SARS-CoV-2 is essential for the spike protein-mediated formation of syncytia [21], organization of membrane lipids [32], subcellular localization [33], and viral entry [20]. Therefore, targeting the palmitoylation of the spike protein of SARS-CoV-2 offers a promising therapeutic strategy against SARS-CoV-2 infection [34]. Additionally, palmitoylation of the NSP1 protein of alphaviruses is necessary for membrane binding and replication [35,36,37]. However, the effect of palmitoylation on ZIKV or other flaviviruses has not been investigated to date. The initial findings of this study suggested that the inhibition of palmitoylation enhances ZIKV infection, and that the envelope protein is palmitoylated at Cys308. Whether the function of nonstructural proteins NS1 to NS5 is regulated by palmitoylation modification deserves further investigation.

The findings revealed that ZDHHC11 is the primary enzyme that catalyzes the palmitoylation of the ZIKV envelope protein. A previous study demonstrated that the ER-anchored protein, ZDHHC11, positively regulates NF-kB signaling by enhancing the oligomerization of TRFA6 [15]. ZDHHC11 also promotes MITA-IRF3 interactions by binding to MITA via its C-terminal domain to promote type I interferon signaling during infections with DNA viruses, but not RNA viruses [16]. Here, we proposed that ZDHHC11 also exerted anti-viral effects upon ZIKV through binding to the envelope protein and palmitoylating the envelope protein. Considering that ZDHHC11 is a multifunctional protein, it may be possible that it limits ZIKV infection by other cellular processes besides palmitoylating envelope proteins.

## 5. Conclusions

We performed a preliminary investigation of the effect of palmitoylation on ZIKV infection, and the findings revealed that the ZIKV envelope protein is palmitoylated at Cys308. ZDHHC11 was identified as the predominant enzyme that interacts with and mediates the palmitoylation of the ZIKV envelope protein. Functional analysis revealed that the overexpression of ZDHHC11 inhibited ZIKV infection, while ZDHHC11 knockdown enhanced viral infection. Overall, our study describes a novel mechanism of host regulation of ZIKV infection and identified potential novel targets of ZIKV infection.

## Data Availability

The data supporting the findings of this study are available within the manuscript.
